# COVID-19 among Pregnant Women Delivering in a Tertiary Care Center: A Descriptive Cross-sectional Study

**DOI:** 10.31729/jnma.6768

**Published:** 2022-01-31

**Authors:** Pooja Paudyal, Neeta Katuwal, Suniti Rawal

**Affiliations:** 1 Department of Obstetrics and Gynecology, Tribhuvan University Teaching Hospital, Maharajgunj Medical Campus, Institute of Medicine, Kathmandu, Nepal

**Keywords:** *COVID-19*, *obstetric delivery*, *pneumonia*, *pregnancy*, *reverse transcriptase PCR*

## Abstract

**Introduction:**

Coronavirus Disease 2019 pandemic is raging across the world and has affected pregnant women as well. There is limited information regarding COVID-19 in pregnant women. The study aimed to find the prevalence of COVID-19 among all pregnant women who delivered during the study period in a tertiary care center.

**Methods:**

This was a descriptive cross-sectional study conducted in a tertiary care center from 16^th^ August to 15^th^ November 2020 after obtaining ethical clearance from the Institutional Review Committee of a tertiary care center. All the women who delivered in the hospital during the study period were enrolled and they were subjected to COVID-19 Reverse Transcriptase Polymerase Chain Reaction test. A total of 667 samples were taken using convenience sampling technique. Data were analyzed using the Statistical Package for the Social Sciences version 24 software. Point estimate at 95% Confidence Interval was calculated along with frequency and proportion for binary data.

**Results:**

Among 667 pregnant women, the prevalence of COVID-19 was 47 (7.05%) (5.10-8.99 at 95% Confidence Interval). Though the majority of women were asymptomatic 40 (85.1%), 5 (10.64%) developed mild disease, 1 (2.12%) each had severe and critical COVID-19 pneumonia.

**Conclusions:**

The prevalence of COVID-19 among pregnant women delivering in our center is similar to other studies done in similar settings. In our study, we found that the majority of women had been asymptomatic and were diagnosed on routine testing. Hence, it is important to test all pregnant women before delivery for Coronavirus Disease 2019 irrespective of the presence or absence of symptoms.

## INTRODUCTION

Coronavirus Disease 2019 (COVID-19); caused by severe acute respiratory syndrome coronavirus 2 (SARS-CoV-2) first emerged in Wuhan, Hubei Province, China in November 2019 and was declared a global pandemic in March 2020 by World Health Organization (WHO); has brought the world down to its heels.^1-4^ The pandemic has affected each one of us and has brought our lives to a standstill. Many have lost their dear and near ones to this unforgiving pandemic.

Pregnant women have not been spared either. The effects of this disease in pregnancy are still under study and information regarding the epidemiology and effects of COVID-19 in pregnancy is limited more so in a country like Nepal.

The aim of this study was to find the prevalence of COVID-19 among all pregnant women who delivered during the study period in a tertiary care center.

## METHODS

This was a descriptive cross-sectional study conducted in the Department of Obstetrics and Gynecology of a tertiary care center. The duration of the study was 3 months from 16^th^ August to 15^th^ November 2020. Ethical clearance was obtained from the Institutional Review Committee of the Institute of Medicine. All women who delivered during the study period and who had had the COVID-19 tests were studied. It was hospital protocol to subject all women admitted for delivery in the center to COVID-19 Reverse Transcriptase Polymerase Chain Reaction (RT-PCR) test and all the women who delivered in the hospital during the study period were enrolled. All patients diagnosed with COVID-19 via a nasopharyngeal swab were analyzed. A convenience sampling technique was used.

The sample size was calculated using the following formula:

n = Z^2^ × (p × q) / e^2^

  = (1.96)^2^ × 0.5 × (1 - 0.5) / (0.04)^2^

  = 601

Where,

n= minimum required sample size
Z= 1.96 at 95% Confidence Interval (CI)
p= prevalence of COVID-19 among all pregnant women who delivered during the study period in a tertiary care center taken as 50% for maximum sample size calculation
q= 1-p
e= margin of error, 4%

Adding a non-response rate of 10%, the final sample size was 662. However, a total of 667 samples were taken.

Our study participants were the pregnant women with laboratory-confirmed SARS-CoV-2 infection on an RT-PCR assay performed on a nasopharyngeal swab before or during delivery hospitalization. Data were collected from patient files regarding age, parity, occupation, and travel history, history of exposure, symptoms, and treatments received if any were also recorded. Other variables studied were comorbidities associated, mode of delivery, maternal and perinatal outcomes.

The criteria for classifying COVID-19 positive patients into severity grades are as follows:

1. Asymptomatic: Those who reported being in usual health without signs or symptoms of COVID-19.^3,4^
2. Mild COVID-19: Symptoms requiring no additional oxygen supplementation or treatment beyond standard labor and delivery care.^3,4^
3. Severe COVID-19: Dyspnea, respiratory rate 30 breaths per minute or higher, oxygen saturation 93% or less on room air, or findings consistent with pneumonia on chest X-ray, or a combination of these.^3,4^
4. Critical COVID-19- any or all of the following: respiratory failure (need for intubation and invasive ventilation), septic shock, and multiple organ dysfunction or failure.^3,4^

Data were analyzed using the Statistical Package for the Social Sciences version 24 (SPSS) software and results were presented in appropriate tables. Point estimate at 95% Confidence Interval was calculated along with frequency, proportion, mean and standard deviations for binary data.

## RESULTS

Among 667 women, who delivered in our hospital during the study period, the prevalence of Coronavirus Disease 2019 was 47 (7.05%) (5.10-8.99 at 95% Confidence Interval). The mean age of the COVID-19 positive women was 29±4.438 years varying from a minimum of 20 years to a maximum of 35 years, and the majority 27 (57.4%) were multigravida. Regarding occupation, most were homemakers 36 (76.60%), followed by five health care workers (nurse 4, doctor 1) (Table 1).

**Table 1:** Maternal characteristics (n = 47).

Parameters	n (%)	Mean±SD
Age (in years)		29±4.438
20-24	9 (19)	
25-30	15 (32)	
>30	23 (49)	
Parity		1.57±0.5
Primigravida	20 (42.5)	
Multigravida	27 (57.4)	
Occupation		
Homemakers	36 (76.6)	
Health care workers	5 (10.6)	
Business owners	2 (4.26)	
Shopkeepers	2 (4.26)	
Service	1 (2.13)	
Teachers	1 (2.13)	

The average duration of hospital stay was 4.89±3.661 days (minimum=1 day; maximum=17 days).

Of the 47 women; only 5 (10.64%) had symptoms when they were tested, the rest 42 (89.36%) were diagnosed on screening at the time of admission for delivery; while 2 (4.2%) developed symptoms postdelivery. Similarly, 5 (10.6%) had a history of exposure (one exposed from husband, one from a coworker, and three others were health care workers) and only three (6.38%) reported travel history, rest 44 (93.62%) COVID-19 positive pregnant women reported no travel history (Table 2).

**Table 2:** Symptoms, exposure, and travel history (n = 47).

Parameters	n (%)
Asymptomatic	40 (85.1)
Symptomatic	7 (14.9)
Fever	4 (8.51)
Fever + cough	1 (2.12)
Fever + cough + shortness of breath	2 (4.25)
**History of exposure**	
Yes	5 (10.6)
No	42 (89.4)
**History of travel**	
Yes	3 (6.38)
No	44 (93.6)

Twenty-three (49%) had some associated comorbidity, most commonly hypertension in 8 (34.7%) and diabetes in 5 (21.7%). Others were hypothyroidism 5 (21.7%), Rheumatic Heart Disease 2 (8.7%), obesity 2 (8.7%), obstetric cholestasis 1 (4.3%), Takayasu arteritis 1 (4.3%) and thrombocytopenia 1 (4.3%).

Though the majority 40 (85.1%) of the women were asymptomatic, 5 (10.6%) developed the mild disease, 1 (2.12%) had severe disease and 1 (2.12%) developed critical COVID pneumonia. All patients recovered and there was no mortality (Table 3).

**Table 3:** Maternal outcome (n = 47).

Parameters	n (%)
**Morbidity**	
Mild disease	5 (10.6)
Severe COVID disease	1 (2.12)
Critical COVID disease	1 (2.12)
**Interventions in severe/critical disease**	
ICU/HDU^*^ admission	2 (4.25)
Non-invasive ventilation	1 (2.12)
Invasive mechanical ventilation	1 (2.12)
Remdesivir	2 (4.25)
Convalescent Plasma Therapy	1 (2.12)
**Mortality**	-

* *ICU-Intensive Care Unit, HDU-High Dependency Unit

The mean gestational age at COVID-19 diagnosis was 37.83±2.24 weeks (minimum= 28.57 weeks; maximum= 40.71 weeks). The mean age of gestation at delivery was 38.38±2.24 weeks (minimum= 28.71 weeks; maximum = 41 weeks). There were 8 (17%) preterm births and one twin set. The mode of delivery was decided as per obstetric indications and only one patient underwent emergency cesarean Section (CS) at 37 weeks for worsening critical COVID19 pneumonia. Out of 47, only 4 (8.5%) delivered vaginally, rest 43 (91.5%) had CS births. Eleven (23.4%) had elective CS and 32 (68.08%) had emergency surgery (Table 4).

**Table 4:** Delivery details (n = 47).

Parameters	n (%)
**Term**	39 (83)
**Preterm**	8 (17)
28-32 weeks	2 (4.25)
34-36 weeks	2 (4.25)
36-36^+6^ weeks	4 (8.51)
**Mode of delivery**	
**Vaginal Delivery**	4 (8.5)
**Cesarean Section**	43 (91.5)
Elective Cesarean Section	11 (23.40)
Emergency Cesarean Section	32 (68.08)

There were 48 babies born to the 47 COVID-19 positive women including one twin set. There were no intrauterine fetal deaths (IUFD), neonatal deaths (NND), or stillbirths. There were 9 (18.7%) babies with low birthweight. Three babies (6.25) had low APGAR scores at one minute while 2 (4.17%) of them had improved APGAR at five minutes; one baby born to the mother with critical COVID-19, still had low APGAR even at five minutes. The baby was diagnosed to have perinatal depression and was admitted to the Neonatal Intensive Care Unit (NICU), intubated and mechanically ventilated but made a good recovery. There were two others needing admission to NICU for Respiratory Distress Syndrome (RDS) and Meconium Aspiration Syndrome (MAS) while three (6.25%) babies were admitted to Neonatal Unit (NNU) for transient tachypnea of the newborn (TTN), RDS, and supportive care for the preterm baby born at 28 weeks (Table 5).

**Table 5:** Fetal outcome (n = 48).

Parameters	n (%)
**Low Birth Weight**	9 (18.7)
<1000gm	1 (2.12)
1000-1500gm	1 (2.12)
1500-2000gm	1 (2.12)
2000-2500gm	6 (12.76)
**Low APGAR score**	4 (33.33)
1 minute	3 (6.25)
5 minutes	1 (2.12)
**NNU admission**	3 (6.25)
Transient tachypnea of the newborn	1 (33.33)
Respiratory Distress Syndrome	1 (33.33)
Supportive for Intrauterine Growth Restriction	1 (33.33)
**NICU admission**	3 (6.25)
Respiratory Distress Syndrome	1 (33.33)
Meconium Aspiration Syndrome	1 (33.33)
Perinatal depression	1 (33.33)
**Intrauterine Fetal Death /Stillbirth**	-
**Neonatal Death**	-

## DISCUSSION

The effects of COVID-19 during pregnancy are a burning concern as the disease is showing no signs of abating any time soon. Literature on COVID-19 in pregnancy is evolving each day-some have shown poor outcomes, some have reported favorable outcomes while others found no definite difference in COVID-19 course in pregnant as compared to non-pregnant subjects.^5-9^

Most of the women in the present study were asymptomatic (85.1%) and were diagnosed on screening at admission at the time of delivery. Fever was the commonest symptom, with all 7 (14.9%) symptomatic patients having fever. Chen, et al. and Nambiar, et al. reported similar results; most patients were asymptomatic and the commonest symptoms were fever, cough, and fatigue.^6,7^ Majority had no travel or exposure history pointing towards community spread of the disease, similar to study in South India by Nambiar, et al. and Shah, et al. from Gujrat.^6,10^

Five women (10.6 %) had mild disease, 1 (2.12%) developed the severe disease, and another 1 (2.12%) developed the critical disease. Guan, et al, in a study from China reported a severe disease rate of 8% in their pregnant population as compared to 15.7% risk of severe disease in the general population.^5^ Shah, et al. also reported that only 7 of 125 (5.6%) pregnant women experienced severe COVID-19 infection.^10^ It is postulated that relative suppression of cell-mediated immunity in pregnancy may be responsible for milder symptoms in COVID-19-positive pregnancies though adequate evidence is not available to conclude the definite effect of this virus in pregnancy.^11^ However, Khoury, et al. have reported a higher rate of severe disease in 16.2%.^12^

Some studies showed increased rates of preterm labor.^13,14^ Khoury, et al. reported 14.6% preterm births whereas Chen, et al. reported that four of the nine patients had preterm delivery but the preterm delivery was not associated with COVID-19 pneumonia.^7,12^ In our study majority delivered at term with only 8 (17%) preterm births and those were unrelated to COVID-19.

The high Cesarean Section rate of 91.5% in COVID-19 positive patients in our study is double the institutional rate of 45% during the same period for non-COVID-19 patients. In a study by Nambiar, et al. there was a similarly disproportionate increase in the rate of cesarean sections (52.28%) when compared to an institutional rate of 23% in non-COVID-19 pregnancies.^6^

The delivery policy for COVID-19 patients in our hospital was as per the Royal College Of Obstetricians and Gynecologists (RCOG), which recommends that the mode of delivery be determined primarily by obstetric indications.^15^ The high CS rates could be attributed to a higher proportion of high-risk pregnancies with associated comorbidities in our study and an increased number of repeat Cesarean Sections.

All the newborns did well in our study. None of the babies developed any symptoms of COVID-19, similar to the study by Nambiar, et al.^6^ The babies were not tested for COVID-19 as the protocol was to test babies only if they became symptomatic. Mothers were allowed to room in with the babies and allowed to breastfeed with strict hand wash instructions and wearing a mask every time. This was in accordance with the RCOG, which recommends against the routine separation of COVID-19-affected mothers and their babies, and WHO guidelines, which states that 'the benefits of skin-to-skin contact and breastfeeding substantially outweigh the potential risks of transmission and illness associated with COVID-19 infection'.^15,16^ Only the babies of the two sick mothers were put in the care of their family members till their mothers were in ICU.

The limitations of the study were the small sample size, short duration, and single center. Neonates were not tested for COVID-19; hence we failed to shed any light on the chances of vertical transmission.

## CONCLUSIONS

The prevalence of COVID-19 among pregnant women giving birth in our study is similar to other studies done in similar settings. In our study, we found that the majority of women had been asymptomatic and were diagnosed on routine testing. Of the symptomatic ones, most had only mild diseases that required no special treatment, however, we must watch out for severe cases since they may deteriorate rapidly. Our study stresses the importance of testing all pregnant women before delivery for COVID-19 irrespective of the presence or absence of symptoms.
